# Abstract of Meteorological Observations Made at Philadelphia, Pa., for the Year 1853

**Published:** 1854-02

**Authors:** J. A. Kirkpatrick


*Abstract of Meteorological Observations made at Philadelphia, Pa., for the year* 1853. By Prof. J. A. Kirkpatrick.

| Month. | Barometer reduced to 32° F. | | | | Thermometer. | | | | Dew Point 2 P. M. | | | | Latitude 39° 57′ 28″ N. Longitude 75° 10′ 40″ W. | | |
|---|---|---|---|---|---|---|---|---|---|---|---|---|---|---|---|
| | Mean. Inches. | Highest. Inches. | Lowest. Inches. | Range. Inches. | Mean. deg. | Highest. deg. | Lowest. deg. | Range. deg. | Mean. deg. | Highest. deg. | Lowest. deg. | Range. deg. | Rain and Snow. Inch. | Wind. Points. | Remarks. |
| January, | 29.971 | 30.709 | 28.920 | 1.789 | 32.02 | 52 | 9 | 43 | 29.32 | 43.3 | 15.7 | 27.6 | 1.84 | N.W.,W.,N.E. | Warmest |
| February, | 29.946 | 30.343 | 29.065 | 1.278 | 35.99 | 59½ | 15 | 44½ | 35.75 | 58.3 | 24.7 | 33.6 | 4.44 | N.W.,W. | day, June |
| March, | 29.873 | 30.345 | 29.320 | 1.025 | 41.80 | 70 | 17 | 53 | 32.67 | 55.7 | 13.0 | 42.7 | 2.46 | WNW,W,SW. | 22d. |
| April, | 29.850 | 30.225 | 29.231 | .994 | 52.15 | 79 | 34 | 45 | 40.30 | 58.3 | 26.7 | 21.6 | 3.88 | S.W.,N.E. | M. Temp. |
| May, | 29.888 | 30.286 | 29.489 | .797 | 63.10 | 87 | 44 | 43 | 49.29 | 61.3 | 33.7 | 27.6 | 5.17 | S.W. | 86½ |
| June, | 29.987 | 30.226 | 29.746 | .480 | 73.58 | 97 | 50 | 47 | 54.17 | 65.7 | 38.7 | 27.0 | 1.05 | S.W.,N.E. | Th. highest |
| July, | 29.955 | 30.146 | 29.609 | .537 | 75.11 | 95 | 61 | 34 | 57.58 | 70.3 | 38.0 | 32.3 | 8.63 | N.E.,N. | June 22,97° |
| August, | 29.913 | 30.097 | 29.597 | .500 | 74.85 | 94 | 55 | 39 | 59.31 | 70.0 | 42.7 | 27.3 | 3.08 | S.W. | Coldest day |
| September, | 29.976 | 30.246 | 29.546 | .700 | 68.32 | 91 | 41 | 50 | 54.19 | 67.7 | 34.3 | 22.4 | 4.46 | S.W.,N. W. | Jan. 16th. |
| October, | 29.974 | 30.331 | 29.512 | .819 | 53.21 | 75 | 35 | 40 | 39.91 | 65.0 | 26.3 | 38.7 | 3.47 | SW,NW,WNW. | M. Temp. |
| November, | 30.157 | 30.502 | 29.467 | 1.035 | 47.13 | 64 | 21 | 43 | 42.22 | 61.7 | 25.3 | 35.4 | 2.32 | SW,NW,NE. | 16½. |
| December, | 29.869 | 30.288 | 29.115 | 1.173 | 33.81 | 56 | 11 | 45 | 29.83 | 46.7 | 16.0 | 30.7 | 2.16 | NW,SW,NE. | Th. lowest Jan. 17, 9°. |
| Ann. Mean. | 29.947 | 30.709 | 28.920 | 1.789 | 54.26 | 97 | 9 | 88 | 43.71 | 70.3 | 13.0 | 57.3 | 42.96 | SW,NW,NE. | Barometer. |
| Winter, | 29.965 | 30.709 | 28.920 | 1.789 | 34.60 | 62 | 9 | 53 | 34.20 | 58.3 | 15.7 | 42.6 | 11.46 | N.W.,W. | Highest 30.709 Jan. 28 |
| Spring, | 29.870 | 30.345 | 29.231 | 1.114 | 51.90 | 87 | 17 | 70 | 40.75 | 61.3 | 13.0 | 48.3 | 11.51 | S.W. | Lowest |
| Summer, | 29.952 | 30.226 | 29.597 | .629 | 74.01 | 97 | 50 | 47 | 57.02 | 70.3 | 38.0 | 32.3 | 12.76 | S.W.,N.E. | 28.920 on |
| Autumn, | 30.036 | 30.502 | 29.467 | 1.035 | 56.22 | 91 | 21 | 70 | 45.44 | 67.7 | 25.3 | 42.4 | 10.25 | S.W.,N.W. | Jan. 23d. |

